# Glycolaldehyde-modified proteins cause adverse functional and structural aortic remodeling leading to cardiac pressure overload

**DOI:** 10.1038/s41598-020-68974-4

**Published:** 2020-07-22

**Authors:** Sibren Haesen, Ümare Cöl, Wouter Schurgers, Lize Evens, Maxim Verboven, Ronald B. Driesen, Annelies Bronckaers, Ivo Lambrichts, Dorien Deluyker, Virginie Bito

**Affiliations:** Biomedical Research Institute (BIOMED), Hasselt University, Martelarenlaan 42, 3500 Hasselt, Belgium

**Keywords:** Cardiology, Cardiomyopathies

## Abstract

Growing evidence supports the role of advanced glycation end products (AGEs) in the development of diabetic vascular complications and cardiovascular diseases (CVDs). We have shown that high-molecular-weight AGEs (HMW-AGEs), present in our Western diet, impair cardiac function. Whether HMW-AGEs affect vascular function remains unknown. In this study, we aimed to investigate the impact of chronic HMW-AGEs exposure on vascular function and structure. Adult male Sprague Dawley rats were daily injected with HMW-AGEs or control solution for 6 weeks. HMW-AGEs animals showed intracardiac pressure overload, characterized by increased systolic and mean pressures. The contraction response to PE was increased in aortic rings from the HMW-AGEs group. Relaxation in response to ACh, but not SNP, was impaired by HMW-AGEs. This was associated with reduced plasma cyclic GMP levels. SOD restored ACh-induced relaxation of HMW-AGEs animals to control levels, accompanied by a reduced half-maximal effective dose (EC_50_). Finally, collagen deposition and intima-media thickness of the aortic vessel wall were increased with HMW-AGEs. Our data demonstrate that chronic HMW-AGEs exposure causes adverse vascular remodelling. This is characterised by disturbed vasomotor function due to increased oxidative stress and structural changes in the aorta, suggesting an important contribution of HMW-AGEs in the development of CVDs.

## Introduction

Advanced glycation end products (AGEs) are heterogeneous compounds formed by irreversible glycation of proteins and are abundantly present in our Western diet^1^. AGEs accumulate in the body with increasing age, and their formation accelerates in diabetic patients^2^ and during increased oxidative stress^3^. There is growing evidence showing that AGEs cause vascular dysfunction^4^, which is an important risk factor for the development of cardiovascular diseases (CVDs) such as coronary artery disease, cardiomyopathy and heart failure, leading to high mortality in the growing and ageing diabetic population^5^. AGEs can be distinguished by their ability to form protein cross-links and/or their fluorescent properties. In addition, they can be classified into low-molecular-weight AGEs (LMW-AGEs, < 12 kDa) and high-molecular-weight AGEs (HMW-AGEs, > 12 kDa)^6^. In CVD patients, increased circulating LMW-AGEs and vascular dysfunction are strong predictors of adverse prognosis since they are both associated with an increased incidence of cardiac events, hospitalisation and mortality^7,8^. LMW-AGEs affect vascular function in two ways: (a) by cross-linking extracellular proteins like vascular collagen or (b) by mediating intracellular signalling via the activation of the receptor for AGEs (RAGE) expressed on endothelial cells^9^. In the process of LMW-AGEs-induced vascular dysfunction, excessive generation of reactive oxygen species (ROS) plays a crucial role ^10^. Negative vascular effects of LMW-AGEs have been described in both macrovascular (i.e. atherosclerosis, coronary artery disease and peripheral artery disease) and microvascular (i.e. nephropathy, neuropathy and retinopathy) complications of diabetes^11,12^. Despite optimistic results on the use of anti-AGEs therapies such as aminoguanidine, pyridoxamine, and alagebrium in animal models of increased LMW-AGEs^13,14^, clinical evidence on their safety and efficacy is currently weak, suggesting the involvement of other AGEs entities^15^. Previous research has mainly focused on mature LMW-AGEs compounds such as pentosidine and carboxymethyllysine (CML) or their precursors such as methylglyoxal, while the contribution of HMW-AGEs to cardiovascular dysfunction is largely unknown. However, the importance of HMW-AGEs in cardiovascular pathophysiology is rising since it has been reported that HMW-AGEs, also present in our diet, have a higher pathogenic potential in diabetic patients^16–18^. In this context, we have shown that HMW-AGEs cause cardiac impairment characterised by myocardial hypertrophy and fibrosis in healthy rats^19^. However, whether HMW-AGEs also affect vascular function remains to be determined. To address this research question, we injected rats daily with HMW-AGEs or control solution for 6 weeks and assessed vasomotor function and wall structure of isolated rat aortic rings.

## Methods

All animal experiments were performed according to the EU Directive 2010/63/EU for animal testing and were approved by the local ethical committee (Ethical Commission for Animal Experimentation, UHasselt, Diepenbeek, Belgium, ID 201,858). Rats were group-housed in standard cages with cage enrichment at the conventional animal facility of UHasselt. Rats were maintained under controlled conditions regarding temperature (22 °C) and humidity (22–24%). Water and food (2018 Teklad global rodent diet, Harlan, Belgium) were provided ad libitum*,* and rats were handled daily to reduce stress.

### Experimental protocol

Adult male Sprague Dawley rats (125–150 g) (Charles River Laboratories, L'Arbresle, France) were randomly assigned to daily intraperitoneal (IP) injection with 20 mg/kg/day bovine serum albumin (BSA)-derived AGEs (HMW-AGEs, N = 22) or an equal volume of unmodified BSA (control, N = 22) for 6 weeks. The generation and validation of the HMW-AGEs and control solutions are performed as previously described^19^. Briefly, fatty acid-free and low-endotoxin bovine serum albumin (7 mg/ml) was incubated with glycolaldehyde dimers (90 mM) in sterile phosphate-buffered saline (PBS, pH 7.4) for 5 days at 37 °C. Dialysis cassettes were used to remove unreacted glycolaldehyde against PBS (3.5 kDa cut-off). The solution was concentrated to obtain glycated BSA of high-molecular-weight and filtered to remove pathogens. In parallel, BSA was dissolved in PBS (7 mg/ml) and subjected to the same dialysis procedure to serve as control. Blood sampling was performed via the rat tail artery after 6 weeks of daily injections. Invasive hemodynamic measurements were performed just before sacrifice. Rats were sacrificed with an overdose of sodium pentobarbital (Dolethal, 150 mg/kg IP, Val d' hony Verdifarm, Beringen, Belgium). Rat aortae were excised for the evaluation of aortic vasomotor function and aortic wall structure.

### Hemodynamic measurements

Hemodynamic measurements were performed under 3% isoflurane anaesthesia supplemented with oxygen. A pre-calibrated SPR-320 rat pressure catheter (AD instruments, Spechbach, Germany) connected to a data acquisition system (PowerLab 4/25 T, AD Instruments) was used to measure hemodynamic parameters in the left ventricle (LV) via the right carotid artery, as described previously^19,20^. LV end-systolic pressure (LVESP), mean pressure and end-diastolic pressure (LVEDP) were obtained and calculated with LabChart8 software (AD instruments). Catheter measurements were continued for at least 10 min to ensure stable and reliable recordings.

### Quantification of cGMP levels

Blood samples, collected at sacrifice, were centrifuged at 2000 rpm for 10 min at 4 °C. Plasma was collected and stored at − 20 °C until used for quantification of cyclic GMP (cGMP) levels using a commercially available competitive enzyme-linked immunosorbent assay (ELISA) kit, according to the manufacturer's guidelines (catalogue number 581021, Cayman Chemical, Ann Arbor, MI, USA).

### Assessment of aortic vasomotor function

#### General procedure

The descending thoracic aorta was isolated from sacrificed rats of both groups and placed in ice-cold Krebs solution (in mmol/L: 118.3 NaCl, 4.7 KCl, 2.5 CaCl2, 1.2 MgSO4, 1.2 KH2PO4, 25 NaHCO3, 0.026 EDTA, 5.5 glucose; pH 7.45). The aorta was cleaned of perivascular fat and connective tissue and cut into aortic rings of 3 mm in length. Aortic rings were horizontally mounted between two steel hooks, one of which was fixed and the other connected with an isometric force transducer (MLT 050/A, AD Instruments) and a data acquisition system (PowerLab 4/25 T, AD Instruments). Aortic rings were placed in individual tissue baths containing Krebs solution, maintained at 37 °C and continuously oxygenated. Passive tension was applied, and rings were equilibrated for 1 h. During this period, aortic rings were washed three times for 20 min with fresh Krebs solution and were then precontracted with 10^−7^ mol/L phenylephrine (PE, Sigma-Aldrich, Diegem, Belgium). After reaching a stable plateau phase, 10^−6^ mol/L acetylcholine (ACh, Sigma-Aldrich) was added to the tissue baths to check vessel viability and endothelial integrity. Aortic rings that failed to react upon PE or ACh application were excluded from further experiments. After washing aortic rings three times for 10 min with fresh Krebs solution, vasocontraction and vasorelaxation responses were assessed.

#### Vasocontraction response

Contractile responses were measured in response to cumulative doses of PE (final bath concentrations: 10^−10^ to 10^−5^ mol/L). Dose–response curves were recorded at steady-state after the addition of each concentration of PE. In addition, the PE-induced contraction response of aortic rings was measured at different passive tensions to assess the length-tension relationship. Briefly, a passive tension of 2 g was applied to all aortic rings. Aortic rings were washed three times for 10 min with fresh Krebs solution, and 10^−7^ mol/L PE was added to induce contractile responses until a stable plateau phase was reached. Aortic rings were washed three times for 5 min, and passive tension was adjusted to 4 g and finally to 8 g. Washout and contraction induction were repeated at these passive tensions.

#### Vasorelaxation response

Aortic rings were precontracted with a single dose of PE (10^−7^ mol/L). Relaxation responses to cumulative doses of ACh (final bath concentrations: 10^−10^ to 10^−5^ mol/L) or sodium nitroprusside (SNP, final bath concentrations: 10^−10^ to 10^−6^ mol/L, Sigma-Aldrich) were measured to assess endothelium-dependent and endothelium-independent relaxation, respectively. In addition, ACh-induced relaxation was evaluated in the presence of either 10^−4^ mol/L N(ω)-nitro-L-arginine methyl ester (L-NAME, Sigma-Aldrich), to investigate nitric oxide (NO) synthesis, or 10^−5^ mol/L indomethacin (INDO, Sigma-Aldrich), to investigate the role of cyclooxygenase (COX). Both inhibitors were preincubated 30 min before PE application (10^−7^ mol/L). To determine the contribution of superoxide radicals, ACh-induced relaxation was measured in the presence of superoxide dismutase (SOD, 150 kU, Sigma-Aldrich), also added 30 min before PE precontraction. Dose–response curves were recorded for 4 min after the addition of each concentration of ACh or SNP. Relaxation responses were expressed as the % of relaxation relative to PE-induced precontraction.

### Histological analyses

Transverse sections of 10 µm thick were obtained from paraffin-embedded aortic tissue and stained using the Sirius Red/Fast Green collagen staining kit to assess aortic fibrosis according to the manufacturer's guidelines (catalogue number 9046, Chondrex, Inc., Redmond, WA, USA). After staining, sections were mounted in DPX. Images were acquired using a Leica MC170 camera connected to a Leica DM2000 LED microscope (Leica, Diegem, Belgium). The area of collagen deposition, indicated by red staining, was outlined in at least four randomly chosen regions. The collagen-positive area was normalized to the total aortic area and expressed as % collagen content. Intima-media thickness was measured as the distance from endothelium to media-adventitia transition and expressed in µm. Both collagen deposition and intima-media thickness were quantified with ImageJ^21^.

### Immunohistochemistry

Heat-mediated antigen retrieval was performed in deparaffinized 8 µm aortic tissue sections using citrate buffer (pH 6) for collagen type I, collagen type III, 3-nitrotyrosine and α-SMA staining or Tris–EDTA buffer (pH 9) for osteopontin. After that, sections were washed with PBS and used for either 3,3′-diaminobenzidine (DAB) or fluorescent immunostaining. For DAB immunostaining, endogenous peroxidase was blocked with 30% hydrogen peroxide (H2O2) diluted 1:100 in PBS. Afterwards, sections were washed with PBS and permeabilized with 0.05% Triton X-100 (Sigma). Then, sections were rewashed and protein blocking was performed to limit background staining (protein block serum-free, X0909, Dako). Consequently, sections were incubated with a primary antibody against 3-nitrotyrosine (1:100, mouse monoclonal, Ab7048, Abcam), α-SMA (1:200, mouse monoclonal, Leica, NCL-SMA) or osteopontin (1:200, mouse monoclonal Ab ab166709, Abcam) diluted in PBS for 1 h at room temperature or overnight at 4 °C for osteopontin, followed by three washes with PBS. A biotinylated anti-mouse antibody (1:100, E0413, Dako) and streptavidin-HRP (1:400, P0397, Dako) were applied for 3-nitrotyrosine, both for 30 min at room temperature and with three PBS washes in between. For α-SMA and osteopontin, EnVision™ + Dual Link System-HRP (anti-rabbit/anti-mouse, K4061, Dako) was applied for 30 min at room temperature. As a negative control, no primary antibody was applied to the section. The presence of 3-nitrotyrosine, α-SMA and osteopontin were visualised using 3,3′-diaminobenzidine (DAB). After immunostaining, nuclei were counterstained using haematoxylin and embedded in DPX mounting medium. Images were acquired using a Leica MC170 camera connected to a Leica DM2000 LED microscope. The level of staining was assessed in three or four random fields per section using the colour deconvolution plugin in ImageJ software^21^ and was expressed as % of the total surface area of interest. For immunofluorescent staining, sections were permeabilized with 0.05% Triton X-100, followed by a washing step with PBS and treatment with protein block. Sections were double-stained with primary antibodies against collagen type I (1:100, rabbit polyclonal, Ab34710, Abcam) and collagen type III (1:100, mouse monoclonal, Ab6310, Abcam) diluted in PBS at 4 °C overnight in a humidified atmosphere. As a negative control, no primary antibody was applied to the section. The day after, sections were washed three times in PBS and incubated with goat anti-rabbit Alexa Fluor 555 (1:500, A-21430, Invitrogen) and donkey anti-mouse Alexa Fluor 488 (1:500, A-21202, Invitrogen) secondary antibodies, for 30 min at room temperature. After 3 washes in PBS, nuclei were counterstained with DAPI for 10 min and incubated for 10 min with 0.1% Sudan black in 70% ethanol at room temperature to reduce autofluorescence. Then, sections were mounted in Fluoromount-G mounting medium (00–4,958-02, Invitrogen). Fluorescence signals were imaged in five random fields per section using a Leica fluorescence microscope (DM 4,000 B LED) with the Leica Application Suite X software. The corrected total fluorescence was quantified using ImageJ software^21^ with the following formula: integrated density—(area of aortic tissue × mean fluorescence of background readings). Two operators blinded for group allocation performed the analyses independently.

### Statistical analysis

Statistical analysis was performed using GraphPad Prism (GraphPad software, version 5.01, San Diego, CA, USA). Normal distribution of data was evaluated by the D'Agostino-Pearson normality test. Accordingly, a parametric t-test or non-parametric Mann–Whitney U test was performed to compare the results of both groups. Dose–response curves were fitted by nonlinear regression and were, along with length-tension curves, analyzed with LabChart software (v8.1.13, AD instruments, Spechbach, Germany). Dose–response curves were compared using two-way ANOVA for repeated measures followed by Bonferroni post hoc test. The half-maximal effective dose (EC50) was compared with the extra sum-of-squares F-test. Simple linear regression models were applied to assess the relationship between different parameters. The sample size is presented as 'N' (number of animals) or 'n' (number of aortic rings). Data are expressed as mean ± standard error of the mean (SEM) and with a 95% confidence interval (CI) or as median [75th percentile; 25th percentile] in case data were not normally distributed. *p*  <  0.05 was considered statistically significant.

## Results

### HMW-AGEs injections increase intracardiac pressure

In vivo LV hemodynamic measurements were performed 6 weeks after the first injection of HMW-AGEs or control solution. As shown in Table 1, LVESP (100 ± 4 mmHg [95% CI 90.3 to 109.2 mmHg] vs. 91 ± 3 mmHg [95% CI 85.4 to 96.6 mmHg], *p* < 0.05) and mean LV pressure (41 ± 2 mmHg [95% CI 36.2 to 46.3 mmHg] vs. 36 ± 2 mmHg [95% CI 31.1 to 39.9 mmHg], *p*  <  0.05) were significantly increased in HMW-AGEs-injected animals compared to control. LVEDP was not different between groups (6.3 ± 1.2 mmHg [95% CI 3.6 to 9.0 mmHg] vs. 4.8 ± 1.6 mmHg [95% CI 1.2 to 8.4 mmHg] in the control group).

**Table 1:** LV pressures are increased after 6 weeks of HMW-AGEs injections.

Hemodynamic parameters	Control	HMW-AGEs
LVESP (mmHg)	91 ± 3	100 ± 4*
LVEDP (mmHg)	4.8 ± 1.6	6.3 ± 1.2
Mean pressure (mmHg)	36 ± 2	41 ± 2*

LV cardiac parameters were obtained in control and HMW-AGEs animals (N = 10/group) after 6 weeks of injections. Data are presented as mean ± SEM. *p  <  0.05 vs. control. LV, left ventricular; LVESP, left ventricular end-systolic pressure; LVEDP, left ventricular end-diastolic pressure.

### HMW-AGEs strengthen the aortic contraction

**Figure 1:**
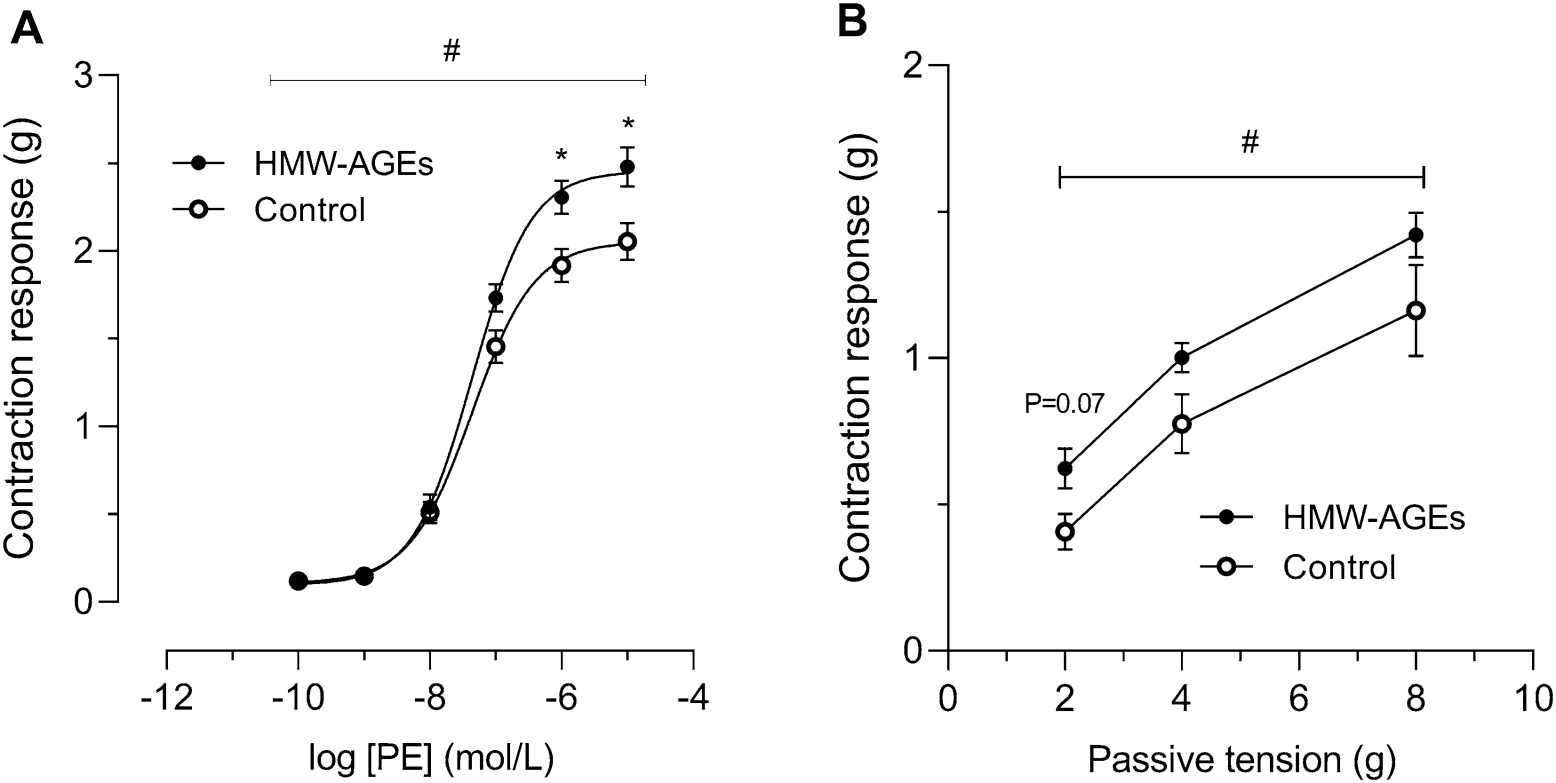
HMW-AGEs strengthen contraction in response to PE. (**a**) Contraction response to cumulative doses of PE (final bath concentrations: 10^–10^ to 10^–5^ mol/L) in aortic rings from 9 control (n_rings_ = 32) and 10 HMW-AGEs (n_rings_ = 38) animals. (**b**) Contraction response to a single dose of PE (10^–7^ mol/L) in aortic rings from 10 control (n_rings_ = 18) and 10 HMW-AGEs (n_rings_ = 18) animals, measured at predefined passive tensions of 2, 4 and 8 g. The contraction response and passive tension are expressed in grams (g) of tension. Data are presented as mean ± SEM. ^#^*p*  <  0.05 HMW-AGEs vs. control (group effect), *p  <  0.05 vs. control (dose effect). PE, phenylephrine.

The vasocontraction response was evaluated by applying cumulative doses of PE to isolated aortic rings from both animal groups. As shown in Fig. 1a, a significant leftward shift of the dose–response curve was observed in the HMW-AGEs group (group effect *p*  <  0.05). Although LogEC50 remained unchanged (− 7.3 ± 0.1 mol/L [95% CI − 7.5 to − 7.2 mol/L] vs. − 7.4 ± 0.1 mol/L [95% CI − 7.6 to − 7.2 mol/L] in the control group; Table 2), vasocontraction was significantly increased at 10^−6^ mol/L (+ 0.39 g [95% CI + 0.02 to + 0.76 g] vs. control, *p*  < 0.05) and 10^−5^ mol/L (+ 0.43 g [95% CI + 0.01 to + 0.84 g] vs. control, *p*  < 0.05) of PE (Fig. 1a). Furthermore, contraction to 10^−7^ mol/L PE was assessed at 2, 4 and 8 g of passive tension to assess the length-tension relationship. Although contractile responses at these predetermined passive tensions were not statistically different (*p* =  0.07), the overall contraction was significantly increased in aortic rings from HMW-AGEs animals (group effect *p*  <  0.05; Fig. 1b).

**Table 2:** Pharmacodynamic characteristics of vasomotor function in rat aortic rings.

	Control	HMW-AGEs
**PE**		
− LogEC_50_ (mol/L)	7.4 ± 0.1	7.3 ± 0.1
E_max_ contraction (g)	2.1 ± 0.1	2.5 ± 0.1*
**ACh**		
− LogEC_50_ (mol/L)	7.4 ± 0.1	7.2 ± 0.1
E_max_ relaxation (%)	92.1 ± 2.1	83.9 ± 2.8
**SNP**		
− LogEC_50_ (mol/L)	8.1 ± 0.1	8.0 ± 0.1
E_max_ relaxation (%)	97.0 ± 1.0	95.2 ± 1.0

The half-maximal effective dose (EC_50_) was derived from dose–response curves generated by nonlinear regression analysis. EC_50_ is expressed as the negative logarithm (−Log EC_50_) of molar concentration (mol/L). Maximal contraction (E_max_ contraction) is expressed in grams (g) of tension. Maximal relaxation (E_max_ relaxation) is expressed as % of PE-induced precontraction (10^−7^ mol/L). PE condition: n_rings_ control = 35, n_rings_ HMW-AGEs = 38; ACh condition: n_rings_ control = 26, n_rings_ HMW-AGEs = 32; SNP condition: n_rings_ control = 28, n_rings_ HMW-AGEs = 28. Data are presented as mean ± SEM. *p  <  0.05 vs. control. ACh, acetylcholine; SNP, sodium nitroprusside; PE, phenylephrine.

### HMW-AGEs impair NO-mediated endothelium-dependent aortic relaxation

**Figure 2:**
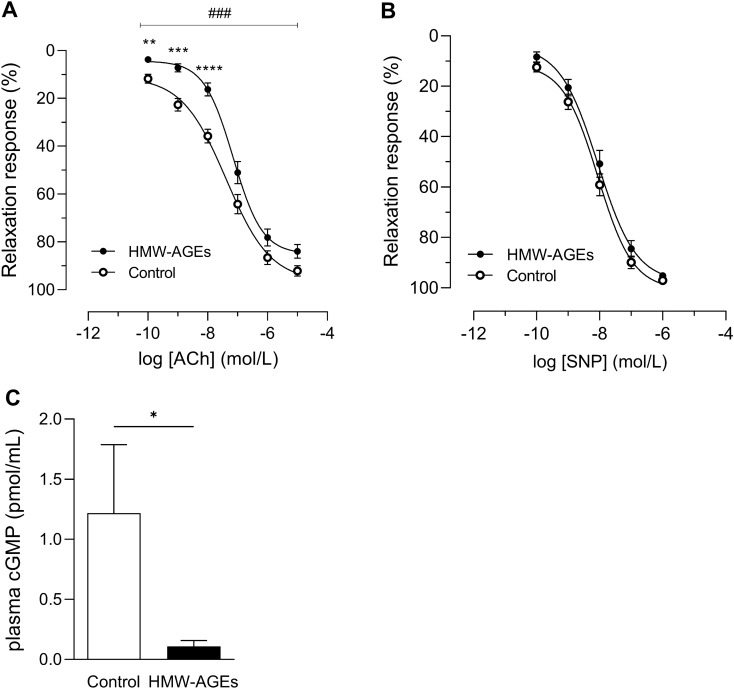
HMW-AGEs impair NO-mediated endothelium-dependent but not endothelium-independent vasorelaxation. (**a**) Relaxation response to cumulative doses of ACh (final bath concentrations: 10^–10^ to 10^–5^ mol/L) in aortic rings from 10 control (n_rings_ = 26) and 9 HMW-AGEs (n_rings_ = 32) animals. (**b**) Relaxation response to cumulative doses of SNP (final bath concentrations: 10^−10^ to 10^−6^ mol/L) in aortic rings of 11 control (n_rings_ = 28) and 8 HMW-AGEs (n_rings_ = 28) animals. The relaxation response is expressed as % of PE-induced precontraction (10^−7^ mol/L). ^###^*p*   <  0.001 HMW-AGEs vs. control (group effect), **p  <  0.01 vs. control, ****p  <  0.0001 vs. control (dose effects). (**c**) cGMP concentration was measured in plasma samples from control (N = 9) and HMW-AGEs (N = 8) animals by ELISA after 6 weeks of injections. *p  <  0.05. Data are presented as mean ± SEM. ACh, acetylcholine; SNP, sodium nitroprusside; PE, phenylephrine; cGMP, cyclic guanosine monophosphate.

After precontraction with 10^−7^ mol/L PE, ACh and SNP induced dose-dependent relaxation in aortic rings in both groups (Fig. 2). In HMW-AGEs animals, ACh-induced relaxation was significantly reduced as demonstrated by a significant rightward shift of the dose–response curve (group effect *p*  <  0.001; Fig. 2a). In addition, relaxation was significantly reduced in response to 10^−10^ mol/L (− 8.0% [95% CI − 13.5 to − 2.4%], *p*  <  0.01), 10^−9^ mol/L (− 15.4% [95% CI − 23.8 to − 7.1%], *p*  <  0.0001) and 10^−8^ mol/L (− 19.5% [95% CI − 30.0 to − 9.1%], *p*  <  0.0001) of ACh compared to control. In contrast to ACh, aorta responsiveness to SNP was comparable in both groups (Fig. 2b and Table 2). As summarized in Table 2, maximal relaxation (Emax relaxation) and LogEC50 for ACh and SNP were not significantly different between groups (Table 2). As a surrogate for NO levels, plasma cGMP concentration was measured. Confirming the aforementioned data, plasma cGMP was significantly reduced in HMW-AGEs animals compared to control (0.1 ± 0.05 pmol/mL [95% CI 0.0 to 0.2 pmol/mL] vs. 1.2 ± 0.6 pmol/mL [95% CI − 0.1 to 2.5 pmol/mL] in the control group, *p*  <  0.05; Fig. 2c).

### SOD reverses HMW-AGEs-induced relaxation impairment

**Figure 3:**
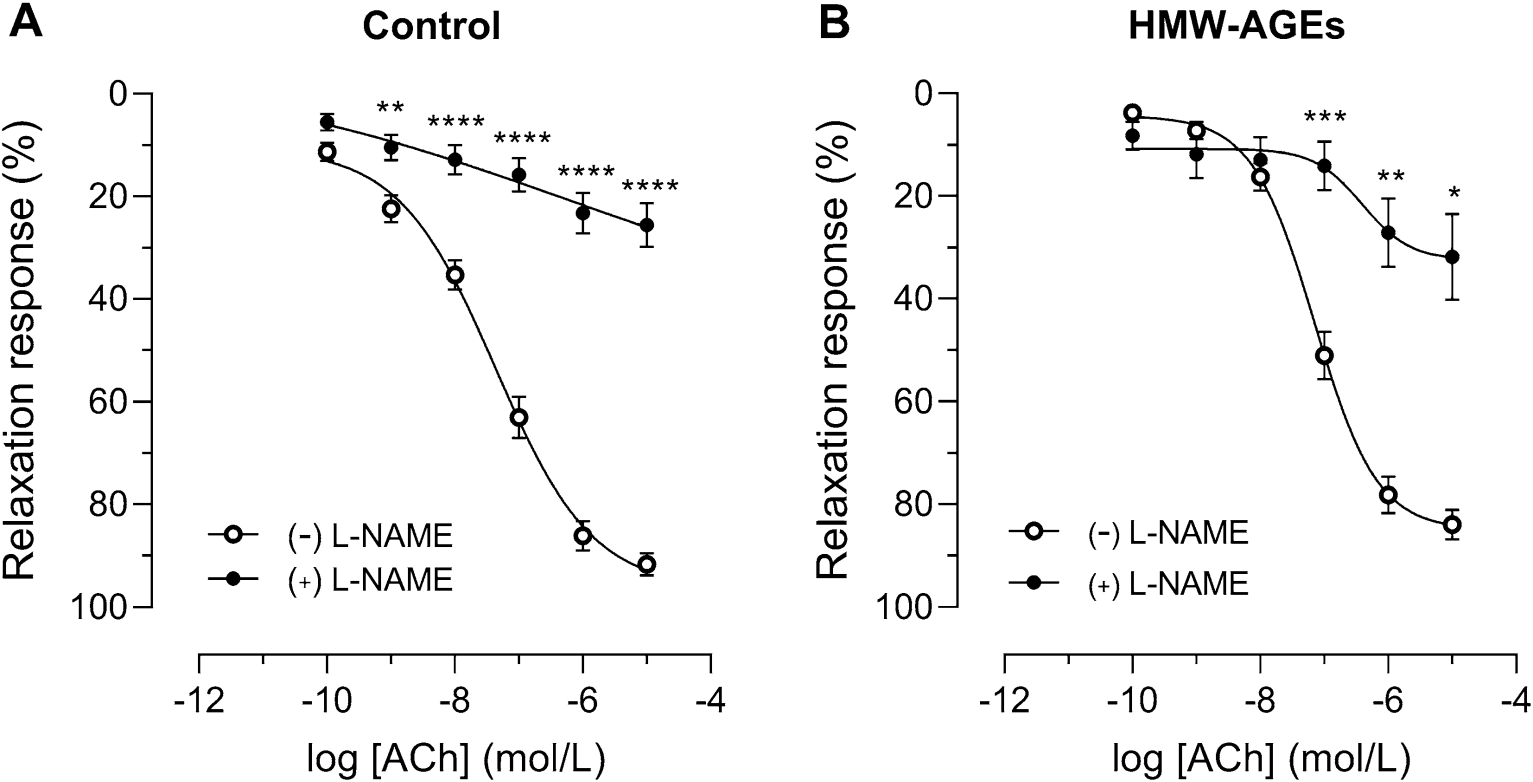
L-NAME inhibits endothelium-dependent relaxation in aortic rings from both animal groups. Aortic rings from control (**a**) and HMW-AGEs (**b**) animals were preincubated with 10^−4^ M L-NAME for 30 min (n_rings_ control = 15 and n_rings_ HMW-AGEs = 5) or left untreated (n_rings_ control = 26 and n_rings_ HMW-AGEs = 32). Relaxation response to cumulative doses of ACh is expressed as % of PE-induced precontraction (10^−7^ mol/L). Aortic rings preincubated with L-NAME were obtained from 9 control and 4 HMW-AGEs animals. Data are presented as mean ± SEM. *p <  0.05 vs. ( −) L-NAME, **p <  0.01 ( −) vs. L-NAME, ***p <  0.001 vs. ( −) L-NAME, ****p <  0.0001 vs. ( −) L-NAME (dose effects). ACh, acetylcholine; PE, phenylephrine; L-NAME, N(ω)-nitro-L-arginine methyl ester.

**Figure 4:**
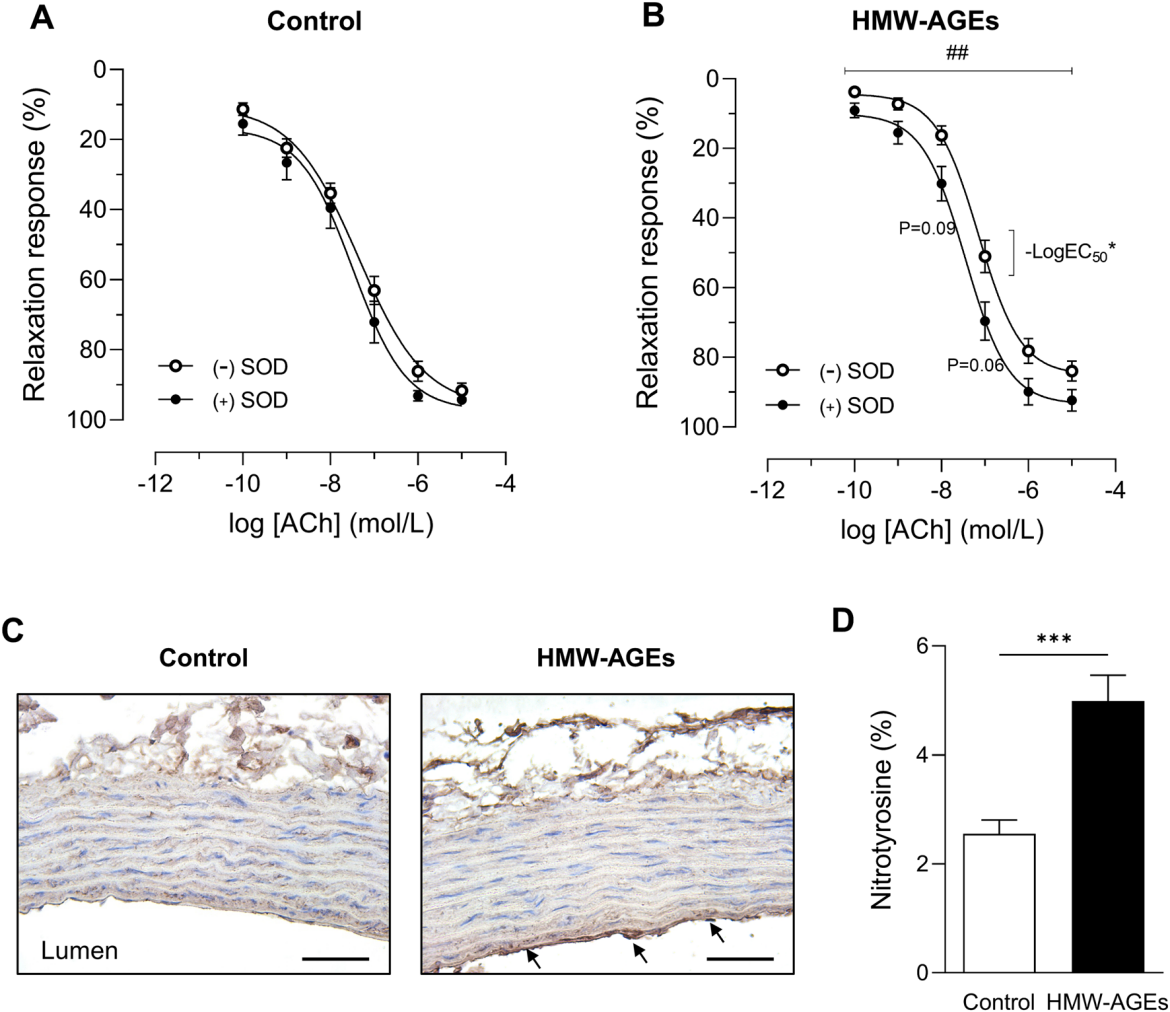
SOD improves endothelium-dependent vasorelaxation in aortic rings from HMW-AGEs animals. A-B. Aortic rings from control (**a**) and HMW-AGEs (**b**) animals were preincubated with 150 kU SOD for 30 min (n_rings_ control = 13 and n_rings_ HMW-AGEs = 22) or left untreated (n_rings_ control = 26 and n_rings_ HMW-AGEs = 32). Relaxation response to cumulative doses of ACh is expressed as % of PE-induced precontraction (10^−7^ mol/L). Aortic rings preincubated with SOD were obtained from 5 control and 6 HMW-AGEs animals. (**c**) Representative images of 3-nitrotyrosine staining in transverse aortic tissue sections from control and HMW-AGEs animals. In the HMW-AGEs group, clear staining of the endothelium (intima) was observed (arrows). Scale bars represent 50 µm. (**d**) Quantification of 3-nitrotyrosine in aortic tissue sections from control (N = 10) and HMW-AGEs (N = 9) animals. Data are presented as mean ± SEM. ^##^*p* <   0.01 ( −) SOD vs. ( +) SOD (group effect), * − LogEC_50_ *p* <   0.05; 7.2 ± 0.1 mol/L for ( −) SOD vs. 7.5 ± 0.1 mol/L for ( +) SOD. ***p <  0.001. PE, phenylephrine; ACh, acetylcholine; SOD, superoxide dismutase.

**Figure 5:**
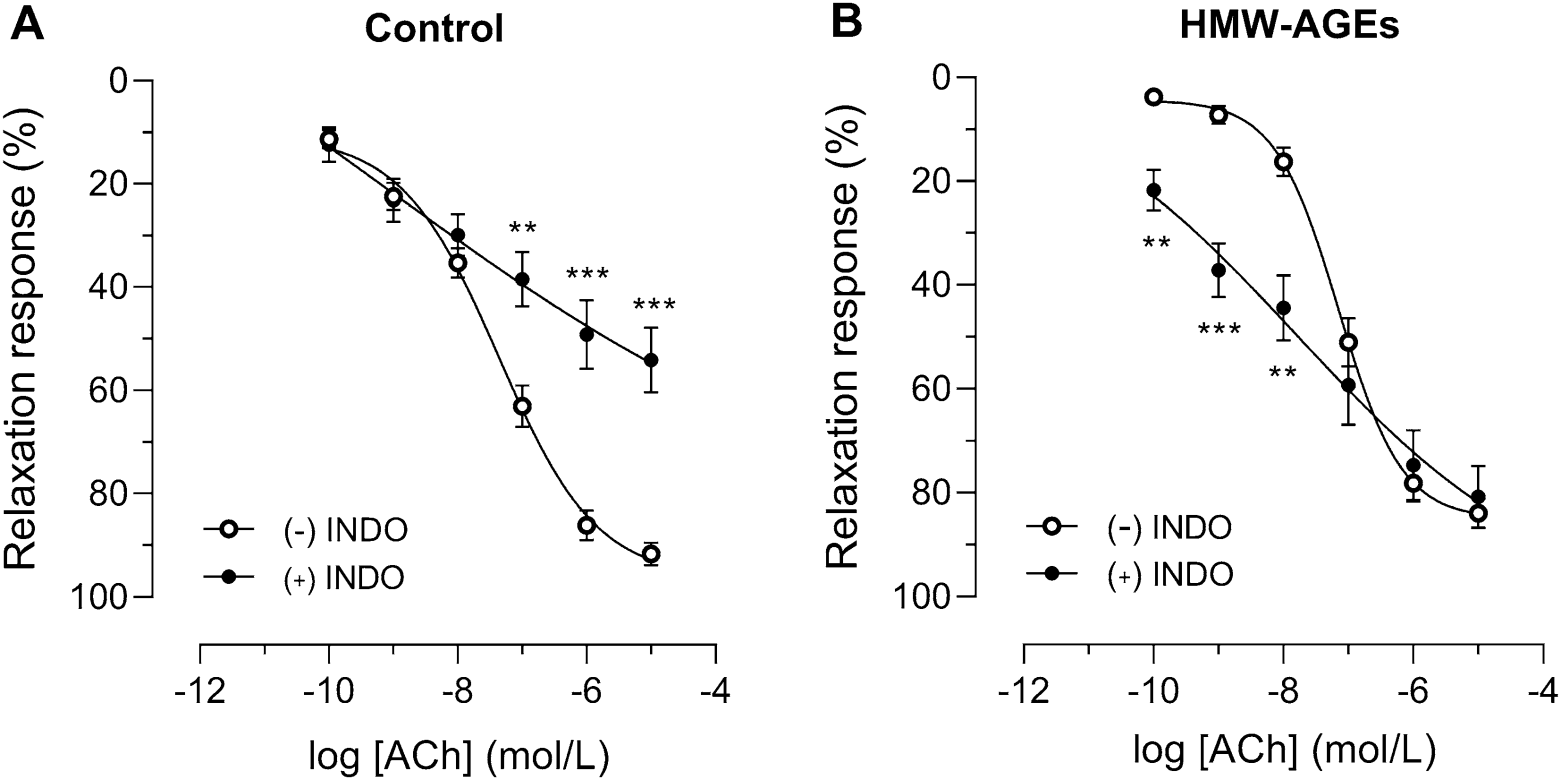
The inhibition of endothelium-dependent vasorelaxation by INDO is less pronounced in aortic rings from HMW-AGEs animals. Aortic rings from control (**a**) and HMW-AGEs (**b**) animals were preincubated with 10^−5^ mol/L INDO for 30 min (n_rings_ control = 12 and n_rings_ HMW-AGEs = 13) or left untreated (n_rings_ control = 26 and n_rings_ HMW-AGEs = 32). Relaxation response to cumulative doses of ACh is expressed as % of PE-induced precontraction (10^–7^ mol/L). Aortic rings preincubated with INDO were obtained from 5 control and 8 HMW-AGEs animals. Data are presented as mean ± SEM. **p <  0.01 vs. ( −) INDO, ***p <  0.001 vs. ( −) INDO (dose effects). ACh, acetylcholine; PE, phenylephrine; INDO, indomethacin.

To unravel the underlying mechanism of HMW-AGEs-induced impairment of NO-mediated endothelium-dependent relaxation, aortic rings from both groups were preincubated with L-NAME, SOD or INDO. Subsequently, dose–response curves to ACh were obtained. L-NAME significantly inhibited relaxation at all doses of ACh to the same extent in both groups (at 10^−5^ mol/L: − 52.1% [95% CI − 15.2 to − 89.0%] in HMW-AGEs, *p* <  0.05 vs. − 66.6% [95% CI − 53.1 to − 80.0%] in control, *p* <  0.0001; Fig. 3A and B). SOD had no effect on ACh-induced relaxation in control animals but significantly increased relaxation in HMW-AGEs animals (Fig. 4a, b). Indeed, a significant leftward shift of the dose–response curve was observed in HMW-AGEs animals (group effect *p* <  0.01). In line with this finding, SOD pretreatment significantly reduced LogEC50 in HMW-AGEs animals (− 7.5 ± 0.1 mol/L [95% CI − 7.7 to − 7.2 mol/L] with SOD vs. − 7.2 mol/L ± 0.1 mol/L [95% CI − 7.3 to − 7.0 mol/L] without SOD, *p* <  0.05), whereas Emax relaxation was not changed (Fig. 4b). To identify whether oxidative stress mediates relaxation impairment in HMW-AGEs-injected animals, 3-nitrotyrosine was measured in aortic tissue sections. Our data show clear staining of 3-nitrotyrosine in the endothelium (intima) of aortic tissue from HMW-AGEs-injected animals (Fig. 4c). Moreover, we measured a two-fold increase in aortic 3-nitrotyrosine staining compared to control animals (5.0 ± 0.5% [95% CI 3.9 to 6.1%] vs. 2.6 ± 0.3% [95% CI 2.0 to 3.1%] in the control group, *p* <   0.001; Fig. 4d). INDO significantly inhibited relaxation in aortic rings from the control group in response to 10^−7^ mol/L (− 25.8% [95% CI − 6.9 to − 44.6%], *p* <  0.01), 10^−6^ mol/L (− 37.4% [95% CI − 15.8 to − 59.0%], *p* <  0.001) and 10^−5^ mol/L (− 38.0% [95% CI − 18.0 to − 58.0%], *p* <  0.001) of ACh, as shown in Fig. 5a. In contrast, no significant differences were observed in HMW-AGEs animals (Fig. 5b). However, INDO-pretreated aortic rings from HMW-AGEs animals showed increased relaxation in response to 10^−10^ mol/L (17.9% [95% CI 30.3 to 5.6%], *p* <  0.01), 10^−9^ mol/L (29.9% [95% CI 46.2 to 13.7%], *p* <  0.001) and 10^−8^ mol/L (28.2% [95% CI 48.3 to 8.0%], *p* <  0.01) of ACh.

### Impaired aortic relaxation in HMW-AGEs animals is related to structural changes in the aortic vessel wall

**Figure 6:**
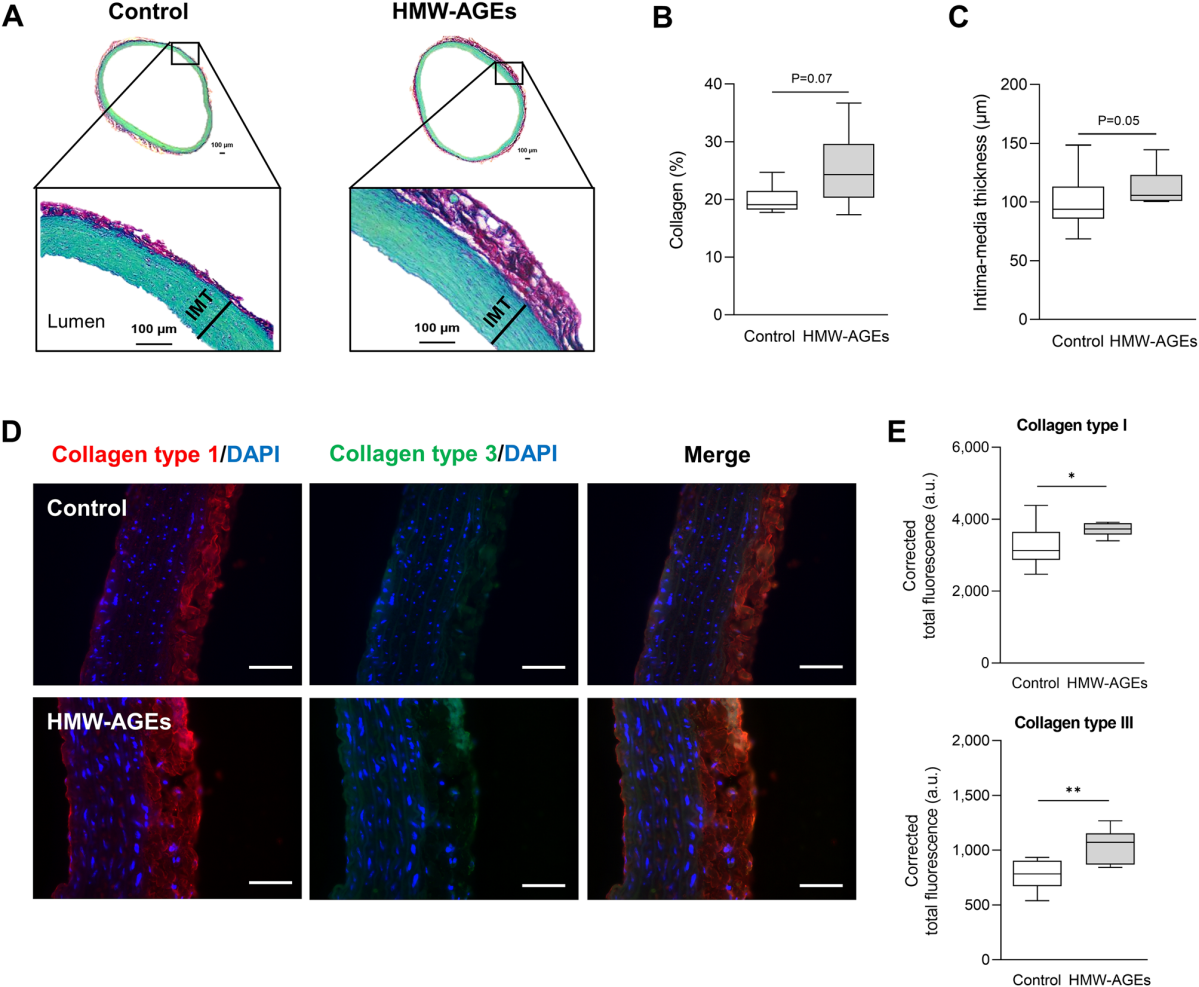
Histomorphometric analysis of the aortic vessel wall. (**a**) Representative images of typical Sirius Red/Fast Green collagen staining in transverse aortic tissue sections from control (left panel) and HMW-AGEs (right panel) animals. Red staining indicates collagen deposition or aortic fibrosis. Intima-media thickness (IMT) is defined as the distance from endothelium to media-adventitia transition. (**b**), (**c**) Quantification of total collagen content (**b**) and intima-media thickness (**c**) in aortic tissue sections from control (N = 6) and HMW-AGEs (N = 7) animals. (**d**) Representative images of immunofluorescence staining for collagen type I (red), collagen type III (green) and DAPI (blue) in transverse aortic tissue sections from control and HMW-AGEs animals. Vessel lumen is located left of the tissue. Collagen type I and type III are mainly present in the adventitia. Scale bars represent 50 µm. E. Corrected total fluorescence of collagen type I (upper panel) and collagen type III (lower panel) in aortic tissue sections from control (N = 9) and HMW-AGEs (N = 6) animals. Data are presented as median [75th percentile; 25th percentile], minimum and maximum. *p < 0.05, **p < 0.01. DAPI = 4′,6-diamidino-2-phenylindole. A.u. = arbitrary units.

The extent of aortic fibrosis was assessed to investigate the role of structural changes in the aortic vessel wall in the impaired relaxation. Representative images of total collagen-stained tissue sections from control and HMW-AGEs animals are illustrated in Fig. 6a. Quantification of collagen deposition revealed that aortic fibrosis tended to be increased in HMW-AGEs animals compared to control animals (24.3 [29.6; 20.3]% vs. 19.1 [21.5; 18.2]% in the control group, *p* = 0.07; Fig. 6b). Moreover, intima-media thickness was increased in HMW-AGEs animals (105.6 [123.0; 100.9] µm vs. 93.8 [113.2; 85.8] µm in the control group, *p* = 0.05), as displayed in Fig. 6c. In addition, we evaluated the abundance of collagen type I and collagen type III in aortic tissue sections through immunofluorescence staining (Fig. 6d). Compared to control, HMW-AGEs-injected animals showed a significant increase in both types of collagen, namely collagen type I (3,730 [3,888; 3,537] a.u. vs. 3,130 [3,647; 2,869] a.u., *p* <  0.05; Fig. 6e, upper panel) and collagen type III (1,071 [1,153; 869] a.u. vs. 783 [935; 672] a.u., *p* <  0.01; Fig. 6e, lower panel). Finally, we evaluated VSMC phenotype through α-SMA and osteopontin staining in aortic tissue sections. HMW-AGEs-injected animals showed a significant increase in osteopontin (24.5 ± 1.4% [95% CI 21.2 to 27.8%] vs. 14.5 ± 1.3% [95% CI 11.6 to 17.5%], *p* <  0.001) compared to control, while there was no statistical difference between groups for α-SMA (*p* = 0.1; Supplemental Fig. 1a, b). Consequently, we observed a significant increase in the ratio osteopontin/α-SMA (0.69 ± 0.06 a.u.[95% CI 0.55 to 0.82 a.u.] vs. 0.37 ± 0.04 a.u.[95% CI 0.29 to 0.45 a.u.], *p* <  0.001; Supplemental Fig. 1c), which was positively correlated with collagen type III deposition (r = 0.59, *p* <  0.05; Supplemental Fig. 1d).

## Discussion

In our study, we show that chronic HMW-AGEs exposure causes intracardiac pressure overload. For the first time, we show that HMW-AGEs alter vasomotor function characterized by strengthened contraction and impaired endothelium-dependent relaxation, which can be attributed to a reduced NO bioavailability and structural changes in the aortic vessel wall.

### HMW-AGEs cause intracardiac pressure overload

For a period of 6 weeks, healthy male Sprague–Dawley rats were daily injected with HMW-AGEs or a control solution of unmodified BSA. Our research group has previously reported an increase in total AGEs levels in the HMW-AGEs group, which validates our animal model^19^. Vlassara et al. (1992) were the first to report that AGEs injections to healthy rats can be used as a model for studying the pathogenicity of AGEs^22^. Other models for AGEs research include those administering AGEs via the drinking water^23^ or via AGEs-enriched diets^24^. However, orally administering a heterogeneous group of AGEs prevents studying the distinct role of the HMW-AGEs fraction independent from LMW-AGEs, which is actually the uniqueness of our study and the results presented here.

Our research group has recently shown that HMW-AGEs injections affect cardiac structure and function, evaluated by conventional echocardiography^19^. To complement these findings, LV hemodynamic measurements were performed. In HMW-AGEs animals, systolic and mean LV pressures are increased, typical features of intracardiac pressure overload. This increase in pressure is likely to be a compensation mechanism to overcome a higher LV afterload, to which increased mean arterial pressure (MAP) or hypertension contributes. MAP can be expressed as (CO × SVR) + CVP, where CO, SVR and CVP stand for cardiac output, systemic vascular resistance and central venous pressure. Since we have previously shown that cardiac output is comparable between HMW-AGEs-injected and control animals^19^, HMW-AGEs are likely to increase SVR being a surrogate for cardiac afterload. As a result, the cardiac muscle becomes hypertrophic, which is in accordance with the previously observed increase in anterior and posterior wall thickness in this animal model^19^. Because systemic vascular resistance mainly depends on the microvasculature, research about the effect of HMW-AGEs on the microvasculature deserves further attention. The increased afterload and subsequent increase of LVESP are likely caused by the enhanced aortic vasocontraction and impaired aortic vasorelaxation, observed in our study. Moreover, the reported changes in aortic wall morphology could suggest that HMW-AGEs decrease vascular compliance by mediating collagen cross-linking^19^. In contrast, LVEDP was comparable between both groups, indicating that diastolic filling is not altered by HMW-AGEs. Previous studies have demonstrated that plasma LMW-AGEs levels (i.e. CML) are significantly higher in spontaneously hypertensive rats as well as in hypertensive patients compared to normotensive controls^25–27^ and that older adults with increased serum CML levels have a higher risk of developing hypertension^28^. Our data suggest that HMW-AGEs, independent from LMW-AGEs, might also contribute to the pathogenesis of hypertension.

### HMW-AGEs disturb vasomotor function in isolated rat aortic rings

In diabetic patients, increased circulating AGEs levels lead to the impairment of endothelium-mediated vasomotor function^29^. In preclinical settings, it is well established that LMW-AGEs have a deleterious impact on vasomotor function but evidence regarding the specific role of HMW-AGEs is currently lacking^22,30,31^. However, recent data demonstrate the rising importance of HMW-AGEs in the development of vascular dysfunction in diabetic patients^16,17^. Therefore, this study investigates the impact of HMW-AGEs on the vasculature and more specific the largest artery supplying the systemic circulation (i.e. aorta).

It has been described that LMW-AGEs and their precursors strengthen vasocontraction in response to the epinephrine analog PE, induced by selective binding to the α1 adrenergic receptor^32,33^. Consistent with these findings, we demonstrate general strengthened PE-induced contraction in aortic rings from HMW-AGEs-injected animals and increased Emax contraction, a measure of agonist efficacy. The increase of aortic contraction might be an extra indication for potential hypertension and increased LV afterload in this animal model, resulting in the intracardiac pressure overload observed in this study. The EC50 value, a measure of agonist potency, was similar in aortic rings from HMW-AGEs and control animals, which suggests that α1 adrenergic receptor sensitivity is not likely to be reduced by HMW-AGEs. Whether HMW-AGEs strengthen contraction by disturbing downstream α1 adrenergic signaling factors like phospholipase C, inositol trisphosphate or calcium deserves further investigation. Results from our research group demonstrated that HMW-AGEs indeed disturb calcium homeostasis in cardiomyocytes^34^. HMW-AGEs possibly disturb calcium homeostasis in endothelial or vascular smooth muscle cells as well, which has been shown before following acute exposure to the AGEs precursor methylglyoxal^32,35^.

The relaxation response to cumulative doses of ACh was assessed to evaluate endothelium-dependent relaxation, a widely used and reliable measure of endothelial function^36^. In this study, we show that HMW-AGEs induce an adverse rightward shift of the ACh dose–response curve, indicating that HMW-AGEs impair endothelium-dependent relaxation and, therefore, endothelial function. As for PE, the EC50 value for ACh-induced relaxation was unchanged. This suggests that aortic sensitivity for ACh, determined by the number and/or performance of muscarinic ACh (M3) receptors on endothelial cells, might not be affected by HMW-AGEs. To investigate vascular smooth muscle performance, we added cumulative doses of SNP to the isolated tissue baths. SNP is a NO donor that mediates direct relaxation of vascular smooth muscle cells without the contribution of endothelial cells. We show that SNP-induced relaxation is similar for both groups, indicating that HMW-AGEs affect endothelial function but do not cause a generalized reduction of vascular smooth muscle responsiveness to vasorelaxant factors. Our data are in line with animal studies examining the effect of LMW-AGEs on the relaxation capacity of isolated rat aortic rings. Chen et al. (2008) have demonstrated that chronic (LMW-)AGEs injections inhibit endothelium-dependent but not endothelium-independent relaxation^37^. Similarly, acute exposure of aortic rings to BSA-derived AGEs impairs endothelial function as evidenced by a reduction of ACh-induced vasorelaxation^38^.

### The underlying mechanism of HMW-AGEs-induced impairment of vascular function

It is well established that NO is the predominant signaling molecule responsible for smooth muscle relaxation and hence vasodilation of blood vessels. NO activates soluble guanylyl cyclase (sGC) which catalyzes the conversion of guanosine triphosphate (GTP) to cyclic guanosine monophosphate (cGMP), the intracellular second messenger that causes relaxation of smooth muscle cells^39^. Since NO is the key mediator of relaxation, it is likely that HMW-AGEs impair endothelium-dependent relaxation by reducing NO bioavailability, as observed before for LMW-AGEs in acute experiments^30^. In this study, we show lower cGMP concentrations in the plasma of HMW-AGEs-injected animals, suggesting indeed a reduced NO bioavailability.

Different mechanisms on how LMW-AGEs reduce NO concentration have been suggested, namely a lower NO production due to decreased endothelial nitric oxide synthase (eNOS) activity and/or NO inactivation secondary to the formation of superoxide radicals^40,41^. To investigate how HMW-AGEs mediate endothelial dysfunction, relaxation in the presence of the NOS inhibitor L-NAME and antioxidant enzyme SOD were measured. Interestingly, SOD restored endothelium-dependent relaxation in aortic rings from HMW-AGEs animals to a similar level of relaxation as in aortic rings from the control group. In addition, we show that the degree of relaxation inhibition by L-NAME was not different between both groups. These data suggest that HMW-AGEs impair aortic vasorelaxation by inactivating or degrading adequately-produced NO compounds through superoxide radicals and oxidative stress, rather than by reducing NO production. Excess superoxide radicals in the vasculature will react with NO, leading to quenching of bioactive NO molecules and the excessive formation of peroxynitrite, which results in the production of nitrotyrosine.^42,43^. As shown by the increased expression of nitrotyrosine in the aortic wall of HMW-AGEs-injected animals, our data suggest an accumulation of peroxynitrite and thus increased oxidative stress. Our findings are of particular interest since experiments performed in diabetic rats have demonstrated that the aortic endothelium is extremely vulnerable for oxidative damage^44^. Indeed, Su et al. (2013) have demonstrated that acute exposure to LMW-AGEs decreases the aortic NO content due to increased superoxide formation^45^. Furthermore, it has been described that LMW-AGEs double the production of ROS and significantly increase nitrotyrosine levels in vascular tissue of rats, resulting in impaired relaxation and increased contraction as also shown in this study^23,46^. In addition, it has been shown that AGEs breaking and antioxidant treatment improves relaxation in mesenteric resistance arteries^47^. Recently, Dobi et al. (2019) reported that BSA-derived LMW-AGEs stimulate excessive ROS production by nicotinamide adenine dinucleotide phosphate (NADPH) oxidases in endothelial cells^48^. The interaction between LMW-AGEs and their receptor RAGE plays a crucial role in this process^10^. In contrast and despite an apparent deleterious effect on cardiovascular function, recent evidence demonstrates that the mechanism of action of HMW-AGEs is not mediated through RAGE activation^19^. A possible mechanism could be, as also suggested in the results of our study, increased oxidative stress and mitochondrial damage. This hypothesis, although not tested in endothelial cells, seems to be confirmed in cardiomyocytes. Indeed, we have recently found that mitochondrial organization is altered in cardiomyocytes from HMW-AGEs-injected animals (unpublished findings), suggesting that HMW-AGEs might indeed increase oxidative stress via mitochondrial damage. Whether a comparable mechanism is involved in endothelial cells remains to be confirmed.

Besides NO, prostacyclin (PGI2) is another relaxing factor secreted by endothelial cells and is formed by COX enzymes during arachidonic acid metabolism^49^. It can be hypothesized that PGI2 is also involved in the inhibition of endothelium-dependent relaxation by HMW-AGEs. Therefore, ACh-induced vasorelaxation was assessed in the presence of the COX inhibitor INDO. Our data demonstrate that INDO significantly reduces relaxation in aortic rings from control animals. However, this effect was smaller than the reduction induced by L-NAME, which suggests that endothelium-dependent aortic relaxation mainly depends on NO in physiological circumstances. Remarkably, the inhibitory effect of INDO on vasorelaxation was less pronounced in aortic rings from HMW-AGEs animals at higher doses of ACh and was even increased at lower doses. This observation can be explained by the fact that superoxide radicals stimulate COX enzymes to produce PGI2^50,51^. This confirms our hypothesis that excess superoxide plays a key role in the impairment of vasorelaxation by HMW-AGEs. Hence, in our study, we can suppose that the inhibitory effect of INDO we observe is blunted by increased PGI2 production secondary to oxidative stress in aortic rings from HMW-AGEs animals. Next to NO and PGI2, endothelial cells are known to secrete endothelium-derived hyperpolarizing factor (EDHF), another vasorelaxant factor, which causes hyperpolarization and thus relaxation of vascular smooth muscle cells, mainly in small resistance vessels^52^. In large vessels such as the aorta however, NO is considered to be the major mediator of endothelium-dependent relaxation and the contribution of EDHF is limited^53^. Nevertheless, the possible effect of HMW-AGEs on EDHF or EDHF-induced relaxation in our model remains to be determined.

Our research group has previously demonstrated that HMW-AGEs induce collagen cross-linking leading to fibrosis in myocardial tissue^19^. Therefore, HMW-AGEs-injected animals are likely to display structural changes in the aortic vessel wall and aortic fibrosis, contributing to the observed impairment of vasomotor function and increased intracardiac pressures. In this study, we show that aortic collagen deposition is higher after 6 weeks of HMW-AGEs injections. More specifically, we observed an increase in collagen type I and collagen type III in HMW-AGEs-injected animals, the predominant subtypes of collagen in artery walls^54^. This increase in interstitial collagen deposition and the content of collagen subtypes has also been observed in the hearts of rats with myocardial infaction (MI)^14^. Interestingly, the increase of collagen deposition with MI was reduced after administering the AGEs-formation inhibitor pyridoxamine. Although our data strongly suggest that HMW-AGEs induce collagen cross-linking in the aortic vessel wall, a possible increase in lysyl oxidase (LOX) expression, a protein that plays a pivotal role in collagen cross-linking, should be confirmed^55^. Overal, our findings are in accordance with previous research indicating that LMW-AGEs contribute to aortic stiffening^22^. Stiffening of central elastic arteries such as the aorta is a characteristic physiological change during aging. However, this process accelerates in diabetic patients with high circulating AGEs levels^56^. Moreover, increased arterial stiffness and reduced compliance are associated with adverse prognosis and mortality^57^. Negative arterial remodeling by LMW-AGEs has also been described in atherosclerosis^58,59^, as LMW-AGEs cross-links are associated with intima-media thickening, which is assessed in clinical practice to detect atherosclerosis^60^. Our data indicate that HMW-AGEs also induce intima-media thickening, suggesting that HMW-AGEs deteriorate arterial health and possibly contribute to atherosclerosis development, which deserves further investigation.

The primary function of VSMCs is to regulate vasomotor tone and thus blood pressure. Mature VSMCs are fully differentiated and exhibit a contractile phenotype with low synthetic activity. However, VSMCs show an extensive plasticity as they can switch their phenotype from contractile to synthetic, also called dedifferentiation^61^. This process has been observed clinically and in animal models of various pathological settings including atherosclerosis^62^, hypertension^63^ and diabetes^64^. In our study, we evaluated the abundance of α-SMA and osteopontin, markers for the contractile and synthetic phenotype respectively^65^. Our data show increased osteopontin/α-SMA ratio in HMW-AGEs-injected animals, which suggests dedifferentiation of VSMCs towards the more secretory phenotype. This increase in osteopontin is implicated in diverse cardiovascular pathologies including atherosclerosis and systemic hypertension^66^. Synthetic VSMCs have high synthetic activity resulting in the production extracellular matrix components^61^. Accordingly, we observed a significant correlation between the osteopontin/α-SMA ratio and collagen type III deposition in aortic tissue sections. Phenotypic switching occurs in response to variations in extracellular signals such as, among others, ROS and oxidative stress^67^. Because nitrotyrosine staining was also present in the aortic media layer of HMW-AGEs animals, next to the intima layer, it can be hypothesized that oxidative stress contributes to VSMC dedifferentiation in our model but remains to be confirmed in future research. Alteration of the phenotype of rat aortic VSMCs has been observed after incubation with LMW-AGEs in vitro^68^ but, to our knowledge, we are the first to show that animals injected with AGEs demonstrate VSMC dedifferentiation in aortic tissue.

In summary, HMW-AGEs induce adverse vascular remodeling as also seen for LMW-AGEs. However, the underlying mechanisms are likely to be different, which is of importance for future therapeutic approaches.

### Limitations

In our study, the intra-aortic pressure was not measured. Even though the intracardiac pressures measured in our study (i.e. LVESP and mean LV pressure), used as surrogates for hypertension and increased cardiac afterload, suggest an increased intra-aortic pressure, this still needs to be confirmed. Moreover, it has to be confirmed whether the increased mean LV pressure is the consequence of increased systemic vascular resistance through HMW-AGEs. Research about the effect of HMW-AGEs on the microvasculature in particular was not performed in our study but deserves further attention. In addition, PGI2 levels were not measured. However, our data of L-NAME and INDO pretreatment clearly indicate that NO is more important for endothelium-dependent aortic relaxation and that NO bioavailability is more likely to be reduced by HMW-AGEs than PGI2. Finally, the administration of a diet rich in HMW-AGEs rather than IP injections with HMW-AGEs would be more clinically relevant. However, such diets are not commercially available and combine LMW- and HMW-AGEs, thus prevent studying the distinct role of the HMW-AGEs fraction. We have previously shown that HMW-AGEs IP injection leads to increased circulating AGEs levels comparable to what we also observed in animal models of diabetic cardiomyopathy^20^ and does not stimulate a general immune response in these animals^19^, further validating the animal model used in this study.

## Conclusion

In conclusion, this study shows that chronic exposure to HMW-AGEs leads to a disturbance of aortic vasomotor function, as illustrated by enhanced contraction and impaired NO-mediated endothelium-dependent relaxation, leading to intracardiac pressure overload. In addition, a reduction of superoxide radicals restores vascular relaxation and nitrotyrosine levels are increased in HMW-AGEs animals, confirming the deleterious role of excess superoxide and oxidative stress. In line with these findings, HMW-AGEs induce morphological changes in the aortic vessel wall with increased aortic fibrosis and VSMC dedifferentiation. These data suggest that HMW-AGEs not only affect cardiac function but also cause adverse vascular remodeling, thereby worsening the prognosis of CVD patients. HMW-AGEs might be key drivers of the ROS-mediated imbalance between pro-contraction and pro-relaxation contributing to a hyper-contractile phenotype, which is a key feature of hypertension. This knowledge creates new opportunities for further research on the specific role of HMW-AGEs in the development and progression of CVDs, as well as CVD risk factors like diabetes, hypertension and atherosclerosis. Strategies to tackle the deleterious effects of HMW-AGEs could lead to better treatment of CVD patients in the future.

### Ethics approval and consent to participate

All animal experiments were performed according to the EU Directive 2010/63/EU for animal testing and were approved by the local ethical committee (Ethical Commission for Animal Experimentation, UHasselt, Diepenbeek, Belgium).

## Supplementary information

Supplementary figure 1.

## Data Availability

The datasets generated and analysed for this study are available from the corresponding author upon reasonable request.
