# Supplementary material for: Glycolaldehyde-modified proteins cause adverse functional and structural aortic remodeling leading to cardiac pressure overload
Source: Sci Rep. 2020 Jul 22;10:12220. doi: 10.1038/s41598-020-68974-4 (PMC7376068; doi:10.1038/s41598-020-68974-4)
Supplement: Supplementary file 1 — Supplementary figure 1. [file 41598_2020_68974_MOESM1_ESM.pdf]

Glycolaldehyde-modified proteins cause Adverse Functional and Structural Aortic Remodeling leading to Cardiac Pressure Overload

Sibren Haesen[1], Ümare Cöl[1], Wouter Schurgers[1], Lize Evens[1], Maxim Verboven[1], Ronald B. Driesen[1], Annelies Bronckaers[1], Ivo Lambrichts[1], Dorien Deluyker[1]*, Virginie Bito[1]*#

[1]Biomedical Research Institute (BIOMED), Hasselt University, Belgium

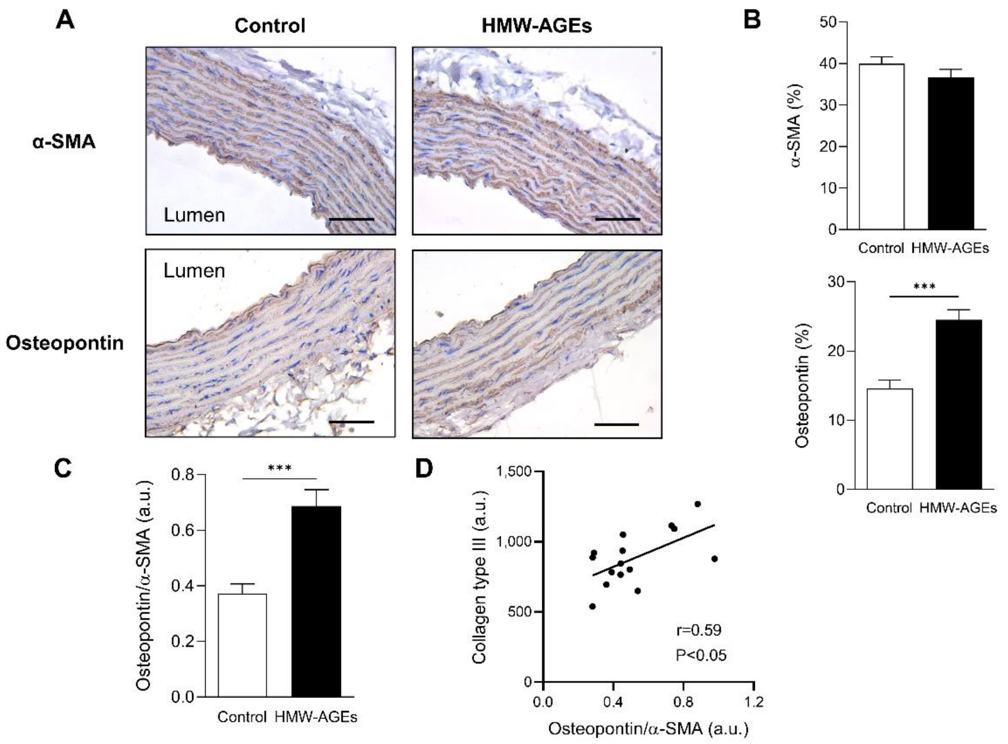

Supplemental Figure 1: HMW-AGEs-injected animals show VSMC dedifferentiation. A. Representative images of α-SMA and osteopontin staining in transverse aortic tissue sections from control and HMW-AGEs animals. B-C. Quantitative analysis of α-SMA (B, upper panel), osteopontin (B, lower panel) and osteopontin/α-SMA ratio (C) in aortic tissue sections from control (N=10) and HMW-AGEs (N=9) animals. D. Correlation between Collagen type III (a.u.) and osteopontin/α-SMA ratio (a.u.) for both groups (N=15). Scale bars represent 50 µm. Data are presented as mean ± SEM. ***$P<0.001$. α-SMA = alpha smooth muscle actin. A.u. = arbitrary units.
